# Plant sexual reproduction: perhaps the current plant two-sex model should be replaced with three- and four-sex models?

**DOI:** 10.1007/s00497-021-00420-5

**Published:** 2021-07-02

**Authors:** Scott T. Meissner

**Affiliations:** Institute of Biology, University of the Philippines Diliman, 1101 Quezon City, NCR Philippines

**Keywords:** Alternation of generations, Fertilization, Mating, Meiosis, Plant sexual reproduction

## Abstract

The two-sex model makes the assumption that there are only two sexual reproductive states: male and female. However, in land plants (embryophytes) the application of this model to the alternation of generations life cycle requires the subtle redefinition of several common terms related to sexual reproduction, which seems to obscure aspects of one or the other plant generation: For instance, the homosporous sporophytic plant is treated as being asexual, and the gametophytes of angiosperms treated like mere gametes. In contrast, the proposal is made that the sporophytes of homosporous plants are indeed sexual reproductive organisms, as are the gametophytes of heterosporous plants. This view requires the expansion of the number of sexual reproductive states we accept for these plant species; therefore, a three-sex model for homosporous plants and a four-sex model for heterosporous plants are described and then contrasted with the current two-sex model. These new models allow the use of sexual reproductive terms in a manner largely similar to that seen in animals, and may better accommodate the plant alternation of generations life cycle than does the current plant two-sex model. These new models may also help stimulate new lines of research, and examples of how they might alter our view of events in the flower, and may lead to new questions about sexual determination and differentiation, are presented. Thus it is suggested that land plant species have more than merely two sexual reproductive states and that recognition of this may promote our study and understanding of them.

## Introduction

In describing the history of the study of plant sexual reproduction (Bennett 1875; von Sachs 1906; Browne 1989; Žárský and Tupý 1995), it is noted how the early model of land plants (embryophytes) as being non-sexual was replaced by a monosexual model in which all these plants were considered to be female but reproducing without the use of fertilization, and then how this view was replaced by what Taiz and Taiz (2017) refer to as the “two-sex model” in which plants were recognized as having distinct male and female sexual reproductive states. This changing in models of plant sexuality led to the opening up of new areas for study. But in order to fit this two-sex model to the land plant life cycle some distinctive judgments had to be made as to which aspects of the plant life cycle were sexual, or not, so that the assumption could be met that these plants had just two sexual reproductive states and no more. Many of these judgments were made well before the discovery that land plants have an alternation of generations life cycle (Taiz and Taiz 2017). Given this, an examination of aspects of plant sexual reproduction from the perspective of the alternation of generations plant life cycle will be the focus of this article.

The discovery that land plant species have an alternation of generations life cycle profoundly changed how we view these plant species, as pointed out by Niklas et al. (2014) and by Friedman (2015). Before this discovery was made, the assumption was that land plant species had a life cycle with just one stage in it with adults (i.e., haplobiontic), suggesting that the study of the adults at that one stage might tell us all about any reproductive or sexual features found in a given land plant species. Thus land plant species were thought to have a life cycle similar to that of animals (Taiz and Taiz 2017). Biologists when considering sexual reproduction in haplobiontic animal species often note that, at this one point in the life cycle where adults exist, there typically are different individual adult animals with distinct sexual states. Thus individual animals are often seen to have just one sexual state each, but most animal species can be characterized as showing two sexual states as these were found in the various adult types present across the entire animal life cycle. Initially plant species were examined assuming a similar life cycle. But with the discovery of the alternation of generations life cycle land plant species were found to have two distinct points in their life cycle with different categories of adults, called sporophytic and gametophytic, making them diplobiontic (Niklas 1997). This implies that a plant species as a whole may have adult individuals in the gametophytic stage of its life cycle that display different sexual reproductive states compared to those states seen in adults in the sporophytic stage of the species’ life cycle. Therefore, with the discovery of the alternation of generations life cycle in land plants we should expand our thinking, to consider the traits of all the adult individuals at each of these stages of the life cycle, if we wish to truly characterize the sexual reproductive features of each plant species as a whole. Another aspect of the alternation of generations life cycle which deserves our attention is the process of sporic meiosis. The argument will be made that in the alternation of generations life cycle sporic meiosis is typically both sexual and reproductive. It will be suggested that, as a consequence of this, new sexual reproductive states are present in land plant species which are not found in typical animal species.

Therefore, while the current two-sex model used for land plants assumes that only the sexual states of male and female are to be present in a given species, this article will question that assumption. Based on an examination of the plant life cycle two new models of plant sexual reproduction (three-sex and four-sex models for homosporous and heterosporous plants respectively) will be presented for consideration. Each of these new models will be compared to the current plant two-sex model in terms of how each accounts for features seen across the land plant alternation of generations life cycle.

The models that we use can summarize existing knowledge and can also set a context for our thinking. It will be suggested that the way the current land plant two-sex model summarizes our knowledge fits poorly with the alternation of generations life cycle. The new three-sex and four-sex models may be better able to describe plant sexual reproduction, doing so in a manner more consistent with this plant life cycle, and with the terminology used for animal sexual reproduction, than does the current plant two-sex model. Furthermore, these new models may offer new ways to view plant sexual reproduction, perhaps suggesting new lines of research, and a few examples of this will be offered. Thus land plant species are suggested to have more than just two sexual reproductive states, and full acceptance of this might help to further advance our studies of plants.

## When are meiosis and fertilization both sexual and reproductive?

To assist an examination of land plant sexual reproduction, the meaning of the terms “sexual” and “reproduction” will first be considered. A sexual process may be taken to be one that has the capacity to produce new genetic combinations (Schwander et al. 2014; Haig 2015). These genetic shifts can be at the genotype level producing new combinations of alleles, or at the genome level as seen with changes in ploidy (Tchórzewska 2017). Both meiosis and fertilization (i.e., syngamy) clearly meet this requirement (Raven et al. 1992; Nelms and Walbot 2019), and here will be accepted as sexual processes in the life cycle of land plant species. In terms of reproduction, the definition given by Kondrashov (1997, pg. 393) would seem relevant: “When a new organism appears from the single cell, each process that produces this cell (mitosis, meiosis, or syngamy) is also a mode of reproduction.” Obviously reproduction by mitotic divisions would be an asexual form of reproduction. However, for either meiosis or fertilization to be reproductive the single cells they produce must grow into new multicellular organisms. Since in embryophytic plants both the zygotes made through fertilization and the spores made by meiosis typically have this ability, both their fertilization and meiosis will be considered to be both reproductive and sexual processes.

**Fig. 1:**
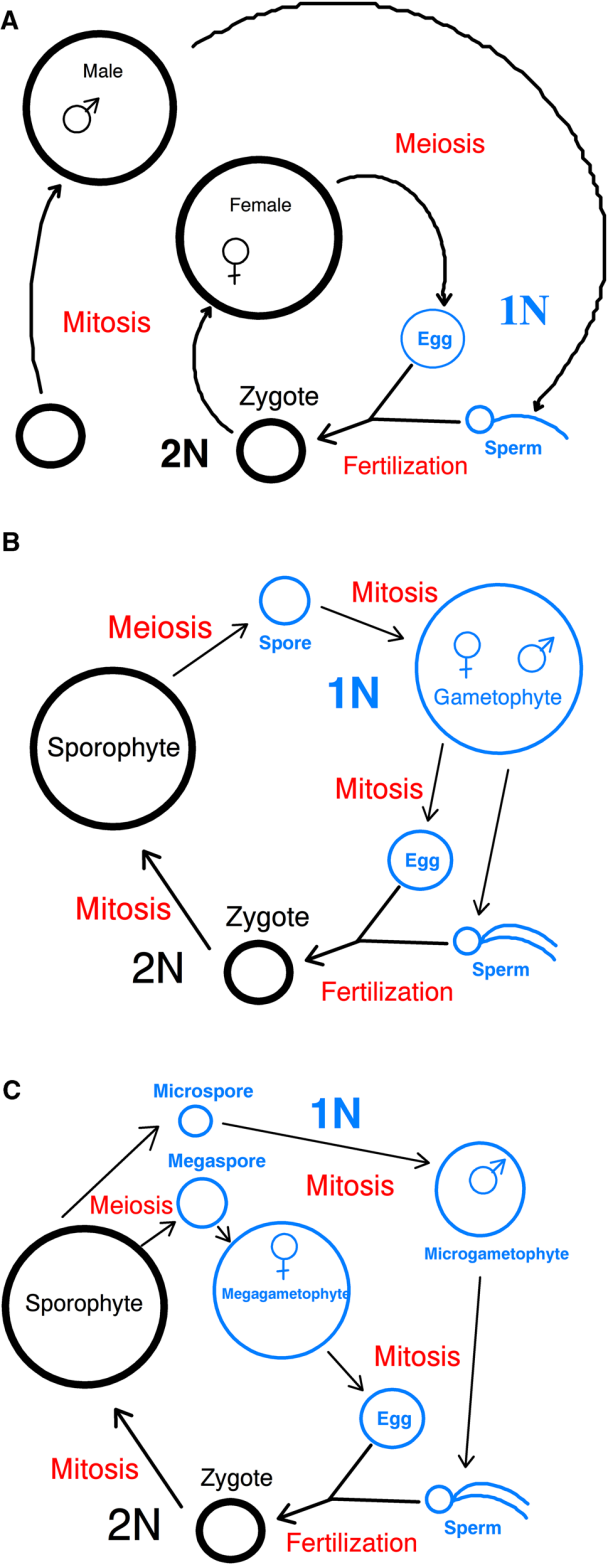
Life cycles of various groups. **a** Typical animal life cycle, showing gametic meiosis. **b** Life cycle of homosporous plant species, such as a fern, with a bisexual gametophyte and a monosexual sporophyte, and; **c** life cycle of heterosporous plant species, such as *Selaginella* sp., with a bisexual sporophyte and two monosexual gametophytes. In each life cycle, the diploid items (2 N) are shown in black, the haploid items (1 N) are in blue, and processes are colored red. Notice that animals use gametic meiosis, while the plants have a distinct gametophytic stage which eventually makes the gametes by mitosis and a sporophytic stage which does sporic meiosis

For contrast and context, let us consider how the above views of sexual reproduction apply to the animal life cycle, as well as review some of the related concepts from animals that are often used in discussions of plant sexuality. Most animal species fit within a two-sex model in a haplobiontic life cycle. Here gametic meiosis is done, followed immediately in the life cycle by fertilization to create a zygote (Fig. 1a). Since the animal’s gametes do not normally individually divide, and so do not become multicellular organisms, gametic meiosis, while sexual, is clearly not a reproductive process for animals. This is not unusual, Hofstatter et al. (2018) note that in many eukaryotic species there are examples of sex and reproduction not being tightly linked, with some eukaryotic taxa displaying sexual processes that are nonreproductive. In contrast, since the animal zygote has the ability to divide mitotically, and so can form a new multicellular individual, for animals fertilization is typically their sole process which is both sexual and reproductive. Notice also that post-zygotic events, such as growth and development of the offspring, its interactions with nurturing parents, and competition with siblings for food*.*, while critical to ensure survival to achieve eventual reproduction by an individual, need not be considered to be sexually reproductive processes themselves as they neither alter the genetic combination nor directly produce new organisms. Thus animal sexual reproduction is often just defined in terms of events closely associated with fertilization. The two adult sexual states are distinguished by the type of gametes made, with organisms that make sperm being called male, and those making eggs being called female. These two sexual states, while typically seen in separate individuals, can exist in one bisexual individual in some animal species (i.e., a hermaphrodite), which may then be capable of self-fertilization (Jarne and Auld 2006). Sexually mature males and females can engage in mating under a variety of animal mating systems (Klug 2011).

### Homosporous plants: the three-sex model

For homosporous land plants (i.e., bryophytes, many ferns and lycophytes), their sexual reproduction will now be described using a three-sex model which arises from the previously described acceptance of both meiosis and fertilization being sexually reproductive processes within the land plant life cycle. For instance, in a homosporous fern species only one type of spore is made, so only one type of sporangium is present in the sporophytic organism. This sporophytic individual is thus sexual in that it has sporangia as its sexual organs in which it does meiosis to make sexual spores (Moore et al. 2016b; Watkins et al. 2016). However, unlike in animals, here meiosis is reproductive as well as sexual since the spores created typically undergo mitosis to create new multicellular gametophytes (Fig. 1b). We can refer to this homosporous fern sporophyte as being a monosexual sporophyte, or simply a sporophyte, and thus easily indicate this sexual reproductive state, which, notice, is neither male nor female as it makes no gametes. These sporophytes are the parents of the subsequent gametophytes that grow from the spores.

The fern gametophytic individuals also show sexual reproduction, as they make the gametes in their organs of antheridia and archegonia (Raven et al. 1992), and these gametes may eventually engage in the sexual process of fertilization. This fertilization is also reproductive as the resulting zygotes are able to grow into new sporophytic individuals. Thus, as Haig and Wilczek (2006) note, here the haploid gametophytes would have the attributes of male and female, being the gamete-forming individuals. In addition, the gametophyte generation of many homosporous plant species, including many ferns, is often bisexual (i.e., hermaphroditic), with the same organism making both sperm and egg gametes. Self-fertilization may occur in such a situation (Klekowski 1973), with the single self here being this individual bisexual gametophyte. Thus these fern gametophytes display a sexual reproductive pattern somewhat similar to that seen in hermaphroditic animals, except that these bisexual gametophytic plants are haploid and their gametes are made by mitosis. Also it may be noted that as ferns are embryophytes, the early growth of the sporophytic organism is supported and nurtured by its gametophytic parent via the archegonium. These gametophytes are thus the parents of the sporophytes.

**Fig. 2:**
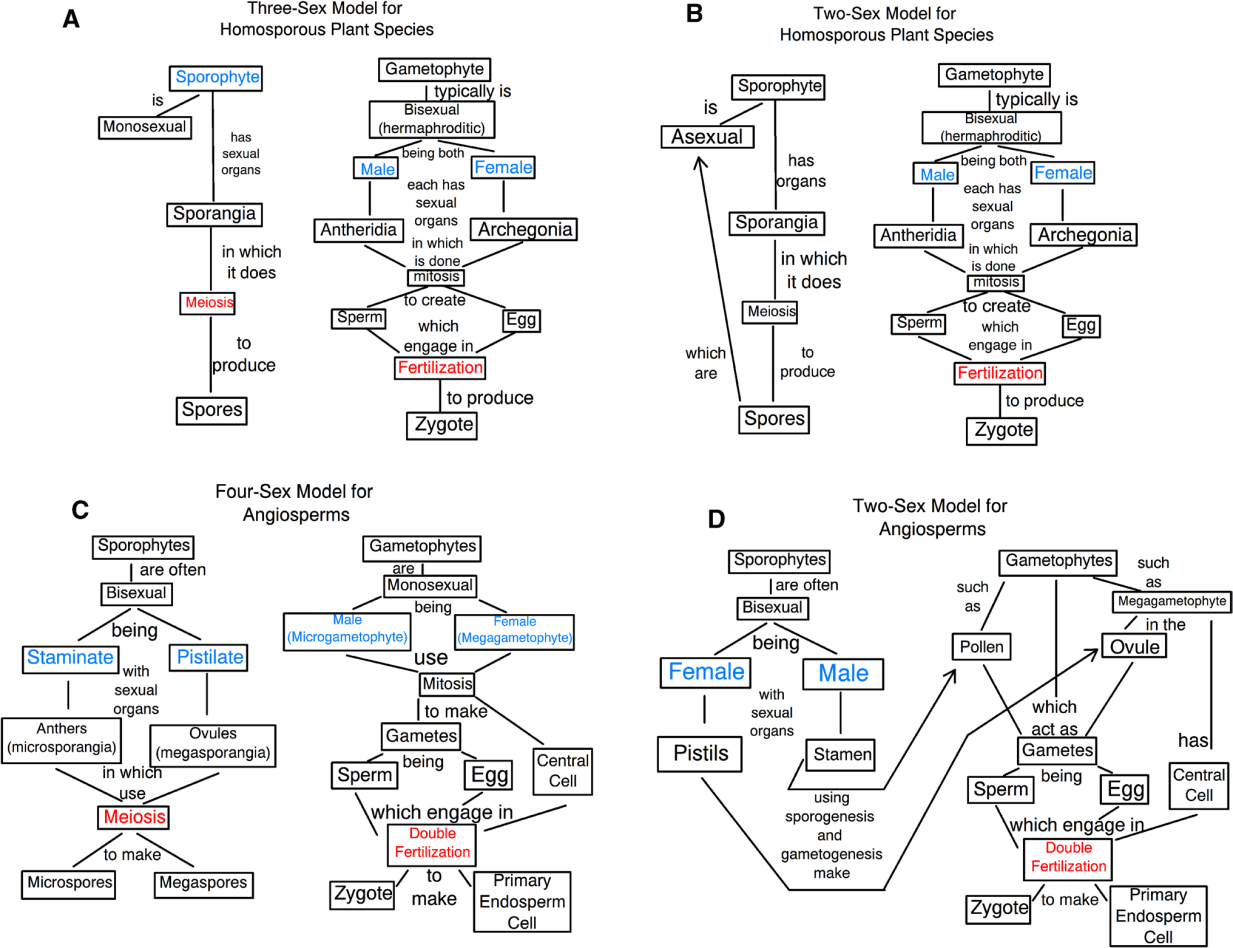
Concept maps illustrating how sexual reproduction is envisioned in the three- and four-sex models versus in the current two-sex model, for land plant species. For each model its accepted sexual reproductive states are indicated in blue, and its recognized sexual reproductive processes are colored red. **a** Homosporous plant species, such as a fern, according to the three-sex model. **b** Homosporous plant species, such as a fern, according to the two-sex model. **c** Angiosperm plant species, such as peas, according to the four-sex model. **d** Angiosperm plant species, such as peas, according to the two-sex model. Notice that the two-sex model accepts only fertilization as a sexual reproductive process, while the three- and four-sex models accept both fertilization and sporic meiosis as sexual reproductive processes resulting in additional sexual reproductive states being present

Thus for this example of a homosporous fern species there are three sexual reproductive states (i.e., a monosexual sporophyte, male and female) displayed in just two individual adults in the life cycle (i.e., a sporophyte, and a bisexual gametophyte) (Fig. 2a). Of course some homosporous plant species have monosexual gametophytic individuals (Lott et al. 2003; Glime 2007; Haig 2016), in which case these three sexual reproductive states would be divided among three distinct individuals. Thus homosporous plant species as viewed under this three-sex model have more sexual reproductive states than do typical animal species.

### Homosporous plants: the current two-sex model

Let us now consider how the current two-sex model is often applied to the life cycle seen in a typical homosporous plant species (Fig. 1b). A critical assumption of the land plant two-sex model is that there are only two sexual reproductive states in the species. Similar to the above three-sex model, the homosporous plant two-sex model assigns to the gametophyte generation the attributes of maleness and femaleness (Haig and Wilczek 2006; Taylor et al. 2007; Haig 2013), and in many homosporous plant species this gametophyte is a bisexual (i.e., hermaphroditic) individual (Fig. 2b). So if it is bisexual then this individual gametophytic organism may often self-mate, and so undergo self-fertilization, to make a zygote. However, with the two sexual states of male and female already assigned, how to refer to the homosporous sporophytic individual in such a species under the two-sex model becomes an issue.

In considering the sporophytes of homosporous land plant species, the two-sex model displays features that result from some interesting judgments. Here, since spore production is not seen in animals, and perhaps because any future sexual state(s) that arise later in the gametophyte cannot be identified at the spore stage, or because with self-fertilization the resulting sporophyte would be presumed to be genetically fully homozygous and so might not contain much genetic diversity, some refer to the spores made by meiosis in the sporangia of homosporous sporophytes as “asexual” in nature (Newton and Mishler 1994; Haufler 2014; Petersen and Burd 2018) (Fig. 2b). Indeed some assign to homosporous plant spores only a role in asexual reproduction (Devos et al. 2011; Mauseth 2017), and the sporangia of homosporous plants, even of vascular species such as ferns, are also sometimes said to be “asexual” structures (Taiz and Taiz 2017), even though meiosis is done within them. Some also suggest that in many bryophytic species the formation of the sporophyte is not a reproductive event as the sporophyte is physiologically dependent on the gametophyte, and so they argue that the sporophyte and gametophyte comprise just one organism (Newton and Mishler 1994), which shows a change in the concept of reproduction in order to fit within the constraints of the two-sex model. Therefore, the homosporous sporophytic individual itself may well have no sexual designation assigned to it under the two-sex model (Raven et al. 1992) (Fig. 2b), leaving all the sexual states of the species to be just the two found in the gametophytic stage of this species’ life cycle.

Thus, while the plant two-sex model typically accepts fertilization as a sexually reproductive process, it does not consider plant meiosis to be a sexually reproductive process (Fig. 2b). Indeed, some early descriptions of the homosporous plant alternation of generations life cycle describe it as an alternation between a sexual generation (i.e., gametophytes) and an asexual generation (i.e., sporophytes) (Harper 1907; Strasburger et al. 1908; Coulter et al. 1910). In this way, the two-sex model manages to view homosporous plant species as having only two sexes, places an emphasis on fertilization, but often ignores sexual reproductive aspects of meiosis and of the sporophyte generation. However, the justification for the two-sex model’s denial of the sexuality of the sporophyte generation in homosporous plant species has largely not been made clear.

### Heterosporous plants: the four-sex model

Now consider the life cycle of a heterosporous non-seed plant species (Fig. 1c), such as *Selaginella* sp., and how this can be described under a four-sex model. This species has a bisexual sporophytic individual. Its sexual organs are the microsporangia and megasporangia, in which sporic meiosis is done to make sexual microspores and megaspores. These spores lead to distinct monosexual male and female gametophytic individuals of this species (Kondrashov 1997). The megagametophyte (female) and microgametophyte (male) each grow endosporicly, bounded by the spore cell wall, producing various cell types (Lyon 1901a, 1901b; Slagg 1932), showing that development of gametophytic organisms need not involve much increase in overall size. Interestingly, in addition to the way the megagametophyte can use its archegonium to support the young growing sporophytic individual, Slagg (1932) notes that for some *Selaginella* species the development of the microgametophytes often begins while still in the microsporangium and suggests that nutrients are passed to it via the tapetum layer of this sporangium to support this early growth. Thus this sporophytic parent may nurture its gametophytic offspring.

Therefore we have in *Selaginella* species four sexual reproductive states (megasporangiate, microsporangiate, male and female) distributed between three adult organisms (bisexual sporophyte, male gametophyte and female gametophyte) across the life cycle. This would be a four-sex condition for the species, and shows how heterosporous plant species can be argued to have more sexual reproductive states than are seen under the current land plant two-sex model, and certainly more than are seen in most animal species.

Next consider the situation in a typical heterosporous seed plant species, such as the pea plant (*Pisum sativum*), an angiosperm. Here the sporophytic individual is bisexual, having two types of sporangia (anther sac/microsporangia in stamen, and ovules/megasporangia in pistils) in which different types of spores are made by meiosis. The two distinct gametophytic individuals that grow from these two spore types create the gametes, and so these gametophytes may be referred to as male (pollen/microgametophyte) and female (embryo sac/megagametophyte) (Fig. 2c). The male and female gametophytes of peas eventually engage in mating interactions to achieve fertilization (Friedman and Williams 2004; Zhong et al. 2019). The synergid cell of the female gametophyte is reported in some species to release a chemical signal that attracts the male gametophyte’s pollen tube to grow toward it (Isogai et al. 2015). Once close enough, the pollen tube cell of the male gametophyte then interacts directly with a synergid cell of the female gametophyte (Mogensen 1978; Boavida et al. 2005), which is done so that sperm delivery can be optimized and quality assured. This would seem analogous, perhaps, with the mating behavior of insemination seen in those animal species that use internal fertilization? In these two examples, the two gamete-forming organisms are engaging in interactions that precede and promote fertilization, and so these may be considered to be mating interactions.

It should be noted that while there may be inbreeding of a lineage if male and female gametophytes that are siblings (i.e., sharing the same sporophytic parent) mate, in this pea species there cannot be any self-fertilization as these gametophytes are two distinct monosexual organisms. Notice also that the bisexual sporophytic pea plant does not make gametes; so while it is sexual it is neither male nor female, therefore this sporophyte cannot be considered to be hermaphroditic. All of this implies that this sporophyte cannot engage in mating or in self-fertilization. This bisexual sporophyte might be referred to as being both staminate (or antherate), as well as carpellate/pistillate (or ovulate) (as in Pelser et al. 2017) (Fig. 2c), or we may say that its flowers are perfect, or complete (Raven et al. 1992), which also conveys this sporophytic bisexual combination clearly. Haig and Wilczek (2006) notes that in angiosperm species all the gametophyte individuals are dioicous (indicating a separation of egg and sperm production between two individuals in the haploid stage of the life cycle) and that the sporophytes are often bisexual. Thus, the pea species has four sexual reproductive states (staminate, pistillate, male, and female) divided among three individuals of this species (a bisexual sporophyte, a male gametophyte, and a female gametophyte) (Fig. 2c).

For other angiosperms species, it may also be noted that dioecious angiosperm species have the staminate and pistillate sexual states divided between two monosexual sporophytic individuals (Haig and Wilczek 2006). Here, under the four-sex model, the four sexual reproductive states are divided between four distinct individual organisms. In contrast, a monoecious angiosperm species would have a bisexual sporophyte, but with distinct staminate and pistillate flowers made in different locations on the body of the same individual plant.

As an interesting side note, it will be recalled that angiosperms carry out double fertilization, and thus both a zygote and a triploid primary endosperm cell are produced (Raven et al. 1992; Sprunck et al. 2012). This primary endosperm cell is thus a product of the sexual process of double fertilization (Friedman and Williams 2004; Friedman 2015) (Fig. 2c), which also may be considered to be a reproductive process in this case as indicated by this cell going on to divide and grow into a multicellular state (Leroux et al. 2014). The endosperm tissue has some interesting functions (Doll et al. 2020), including a role in supplying nutrition to the growing sporophyte (Friedman 1995). But two items of interest for this article are perhaps worth noting. First, while the primary endosperm cell is a product of sexual reproduction, the endosperm multicellular organism it grows into never reproduces itself and never carries out a sexual process. So here is a plant multicellular “body,” if it is accepted as such, that is an example of a non-sexual and nonreproductive organism. An interesting null state, perhaps, for comparison to the individuals in the sporophytic and gametophytic stages of the life cycle which are sexually reproductive. The second item of interest is that, if again one accepts the endosperm to be a multicellular “body,” this implies that while most embryophytic species are diplobiontic in terms of the types of sexually reproductive adult organisms found in their two life cycle stages, those species that carry out double fertilization and produce endosperm may be argued to be triplobiontic in terms of the total number of types of categories of plant bodies present in their life cycle (i.e., sporophytic, gametophytic and endospermic). These two items perhaps indicate just how highly derived are those plant species that carry out double fertilization and create an endosperm.

This new four-sex model, as well as the three-sex model presented earlier, requires that we recognize meiosis as a fully sexually reproductive process, and requires that the sporophytes and the micro- and megagametophytes each be accepted as individual sexually reproductive adult organisms. In other words, we have to accept that the land plant alternation of generations life cycle is an alternation of sexually reproductive generations. Based on the three-sex and four-sex models, therefore, in the life cycle of various species of land plants there are typically either three to four distinct sexual reproductive states distributed between from two to four individual organisms across the life cycle. This is rather different from the limit of just two sexually reproductive states per species that the current plant two-sex model imposes.

## On pollination

The astute reader may have noticed that in the above outline of the four-sex model as applied to angiosperms there was no mention made of pollination. That was because pollination, according to the definitions presented earlier, is neither a sexual nor a reproductive process: Pollination alone does not produce a new organism, nor does it alter the genetic combination or ploidy in any manner. As this view is rather different from the way the two-sex model places pollination into many aspects of sexual reproduction, a brief description of how angiosperm pollination would be viewed under the four-sex model follows.

The angiosperm male gametophyte (pollen) has an early stage of endosporic development within the anther sac, and a later stage of exosporic development on the stigmatic surface and in the stylar tissues of the pistil. In between these two developmental stages, there is a period of relative dormancy during which, by various means, the pollen is moved from the anther sac to the stigmatic surface, a process we call pollination (Raven et al. 1992). An analogous process is perhaps seen in marsupial animals where, after a period of development in the womb, the immature offspring must move to the marsupial pouch to continue its development (Edwards and Deakin 2013). Notice that in some angiosperm species pollination is done before the pollen grain has made its sperm, and even if the sperm are present during pollination they are often not yet sexually mature (Snell 2012; Sprunck et al. 2012; Williams et al. 2014; Liu and Wang 2021). After pollination, the male gametophyte must grow toward the female gametophyte, maturing as it does so, and then, as also noted above, engage in mating interactions before fertilization can be attempted between these two gametophytic organisms (Escobar-Restrepo et al. 2007; Ge et al. 2007, 2017; Sprunck et al. 2012; Stegmann and Zipfel 2017). Thus under the four-sex model, pollination is completed upon pollen arrival at the stigmatic surface, and so is not viewed as being fertilization since this precedes the eventual fertilization process. Recall that in animal species there are many things that must happen for a young animal to survive to eventually reproduce, and yet the early processes of growth and developmental in animals are seen as distinct from those of later sexual reproduction. A similar distinction is being applied here in considering angiosperm male gametophytes undergoing pollination versus later engaging in fertilization.

In addition to not being fertilization, pollination is also not mating. This is because an immature male gametophyte cannot “mate” with sporophytic tissues of the pistil; not just because it is immature but more tellingly because sporophytes never make gametes and so cannot engage in mating. Rather mating is seen as an interaction between two gamete-forming individuals, and so occurs between the angiosperm male and female gametophytes. The parent sporophyte in this case acts to nurture its female gametophytic progeny, and also nurtures the male gametophytes which may be its direct progeny or this pollen may be the offspring of some other sporophytic individuals. These nurturing and other interactions are clearly important (Crawford and Yanofsky 2008), and subject to selection, but are not here considered to be mating. Thus under the four-sex model mating occurs between gametophytic males and females, but not between one generation and another in the plant life cycle; females and males mate with one another, but male gametophytes do not mate with the sporophytic parents of female gametophytes. Thus pollination, which involves the sporophyte and the pollen, is not mating.

Also the four-sex model requires that we adopt the view that self-pollination is different from self-fertilization, as these two processes involve distinct “self” individuals. Self-pollination typically involves one sporophytic “self” and the movement of an immature male gametophyte (pollen) from the anther sac to the stigmatic surface of this single bisexual sporophytic individual; clearly many angiosperm species show self-pollination. In contrast, self-fertilization involves a “self” that must be able to make both egg and sperm, and therefore must be a bisexual gametophyte. A bisexual gametophytic state is seen in many homosporous plant species, including many ferns and bryophytes, but simply does not occur in angiosperms or other heterosporous plant species where the gametophytes are each monosexual. Angiosperm species, therefore, do not have sporophytic individuals or gametophytic individuals that can self-fertilize. However, many angiosperm species, by the ability of their sporophytes to self-pollinate, can produce situations where there will be inbreeding of their line by causing sibling female and male gametophytes, who are the progeny of the same sporophytic parent, to mate later in the life cycle.

Thus, as viewed under the four-sex model, pollination is a process that interrupts distinct periods of development of the male gametophyte, and has no direct analogy in many animal species, other than perhaps with a few animal species such as marsupials as already mentioned. Therefore, while pollination is clearly important in seed plants, and its study of great interest, whatever selective pressures operate to ensure that pollination occurs should perhaps not be considered to be sexual selection? Natural selection obviously applies in many ways in different seed plant species to ensure that pollination is achieved. But only when the male gametophyte and female gametophyte engage in mating interactions, or are directly acting to achieve fertilization, would it seem proper to refer to sexual selection as being involved.

### Heterosporous plants: the two-sex model

Let us next consider how the land plant two-sex model is applied to heterosporous plant species, especially angiosperms. As stated previously, this two-sex model focuses mainly on fertilization as the sole sexual reproductive process (Crawford and Yanofsky 2008), and largely treats meiosis as an asexual process. But to meet its assumption that there are only two plant sexual reproductive states, the two-sex model for seed plants redefines major terms compared to their typical meanings as applied to animals. Some of the terms redefined include: “male,” “female,” “fertilization” and “mating” (Table 1). Also the two-sex model obscures the angiosperm gametophytic individuals so that only the sporophytes are considered as “self.” A brief description of each of these redefinitions follows.

**Table 1:** Meanings of common sexual reproductive terms applied to animals versus their altered meanings used under the land plant two-sex model as applied to angiosperms, compared so as to illustrate how the different meanings can create the potential for confusion. Notice that under the four-sex model for angiosperms the meanings of these terms are similar to that used for animals for these features of sexual reproduction

Terms under each model	Description
*Male*	
Animals and Plant 4-sex model	An individual organism that at sexual maturity makes sperm
Plant 2-sex model	An individual organism that makes pollen, with the concept covering events and structures from microsporogenesis through microgametogenesis
*Female*	
Animals and Plant 4-sex model	An individual organism that at sexual maturity makes eggs
Plant 2-sex model	An individual organism that creates ovules/seeds/fruits, with the concept covering events and structures from megasporogenesis through megagametogenesis and beyond to the setting of fruit
*Mating*	
Animals and Plant 4-sex mode	Interactions between egg- and sperm-forming individuals, which directly precede and promote fertilization
Plant 2-sex model	Often focuses mainly on achievement of pollination and the interactions between an immature microgametophyte and stigmatic/stylar tissues of a sporophyte
*Fertilization*	
Animals and Plant 4-sex model	Union of an egg and sperm to make a zygote
Plant 2-sex model	Often viewed as starting with pollination, and includes pollen tube growth toward the embryo sac and sperm delivery to achieve zygote formation

For angiosperms as viewed under the plant two-sex model the attributes of “male” and “female” are altered so that they no longer apply just to the gamete-forming individuals (Table 1), but extend also to the sporophytes so that stamen are considered to be “male” organs, and pistils “female” organs (Dellaporta and Calderon-Urrea 1993; Boavida et al. 2005; Nores et al. 2015; Charlesworth 2016; Glick et al. 2016; Lankinen et al. 2016; Matsuhisa and Ushimaru 2019; Placette 2020); this is done even though sporophytes never make gametes (Mauseth 2017). Thus the concepts of “male” and “female” used under the two-sex model are broadened so that both microsporangiate sporophytes and microgametophytes fall under this broader redefined term “male,” and so that megasporangiate sporophytes and megagametophytes are covered by this new redefined term “female.” This extends these concepts across the two plant generations (Fig. 2d). In addition there are new functions associated with these new concepts of “male” and “female” that are no longer limited to just gamete formation. The new “male function” includes the production of pollen, while the “female function” is the production of ovules, or seeds, or fruits, and typically both of these new functions are attributed to the sporophytic individuals (Carr 2013; Carper et al. 2016; Harth et al. 2016; Kamath et al. 2017; Christopher et al. 2019) (Fig. 2d). Also under the two-sex model, the pattern of pollination used is often described as a type of “mating system” (Willson and Burley 1983; Etterson and Mazer 2016; Lankinen et al. 2016; Malagon et al. 2019), and sporophytes are said to engage in “mating.” Thus “mating” is apparently redefined so as not to require the ability to make gametes, which is different from how the term is used for animals (Table 1). Furthermore, the process of pollination is often regarded as being a part of “fertilization,” thus extending the concept of “fertilization” to include events from pollination through actual zygote formation (Till-Bottraud et al. 2005; Castilla et al. 2016; Taiz and Taiz 2017) (Table 1). Also under the two-sex model there often is consideration of how some angiosperm species are said to carry out “self-fertilization,” by which it is implied that a “hermaphroditic” angiosperm sporophytic individual “mates” with itself in some fashion (Brewbaker and Natarajan 1960; Barrett 2002; Cruzan and Barrett 2016; Grossenbacher et al. 2016; Beaudry et al. 2020), and so this one individual sporophytic organism is suggested to achieve zygote formation all by itself.

Thus, even though the same terms are being used in this plant two-sex model as are used for animal species, their meanings are altered relative to how they are used for animals (Table 1). The sum of these redefinitions under the two-sex model acts to largely obscure the angiosperm gametophytic individuals and their roles; they are subsumed under the new broader definitions of “male” and “female,” these gametophytes are not acknowledged to be independent “selves,” rather “mating” and “fertilization” become processes the sporophytes carry out by themselves. Further, by referring to pollen in anthers and to the embryo sacs in ovules as the products of “male” and “female” sporophytes, and to how the pollen “fertilizes” the ovules (Jarne and Charlesworth 1993; Moore and Pannell 2011; LoPresti et al. 2018), the gametophytes are said to act as mere gametes (Jesson and Ganock-Jones 2012) (Fig. 2d). This seems to deny these gametophytes a role as distinct organisms in the angiosperm alternation of generations life cycle? Thus the two-sex model obscures the angiosperm gametophyte generation and redefines common terms in significant ways.

Oddly, these terminological alterations (Table 1) under the two-sex model also seem to act to make the life cycle of angiosperms appear in some ways to be similar to that seen in animals, at least by placing emphasis on fertilization as the sole sexual reproductive process as it is in animals, and by presenting an impression that the angiosperm sporophyte is the sole adult individual in the life cycle through downgrading of the gametophytes to the status of mere gametes and assigning their functions to the sporophytic individual. Thus, under the two-sex model, the elegant and sophisticated plant alternation of generations life cycle is treated, in effect, as if it is just like the mundane and simplistic animal life cycle. Whether that is the intent or not is unclear, as seldom are these redefinitions acknowledged in the literature, let alone justified compared to the alternate meanings used for animals. But this illustrates, perhaps, how this two-sex model alters our thinking in that it tends to guide our thinking about seed plant species to make us view them as though they are just like animals.

Taiz and Taiz (2017) do note that while the land plant two-sex model is technically incorrect as applied to angiosperms, they consider it to be simpler. However the simplification produced by these subtle redefinitions of terms (Table 1) under the two-sex model can also create some confusion. For instance, when reference is made to a “male” angiosperm, this redefined “male” concept includes two organisms under that label: So is the intent to refer to the gamete-producing organism (i.e., the microgametophyte), or to the sporophyte, or both? Is an angiosperm sporophytic “male,” which never makes sperm itself, truly comparable to an animal male that does make sperm? They are both being called male so we would likely expect the sporophytic “male” to be similar to an animal male, but the different definitions used seem to indicate that these are very different items (Table 1); why then is the same term applied? Similarly, when angiosperm sporophytes are said to “self-fertilize” does that mean we are to ignore any role of the micro- and megagametophytes in fertilization; are they not their own “selves”? Is the “self-fertilization” said to be done by some angiosperm sporophytes supposed to be similar to the self-fertilization done by a bisexual fern gametophyte, or by a hermaphroditic animal individual? Or is the similarity merely the impression created by using a similar label which turns out to have very different meanings in these different contexts? If so, that seems like a situation ripe for the production of confusion.

These sorts of reconceptualizations can place heavy burdens on those attempting to understand this area of study as it can require that each of these term’s contrasting meanings has to be considered, and often a very careful examination of their use has to be made to discern the intended meaning. However, those who are unaware that the two-sex model imposes these redefinitions may be unable to carry out such examinations. This two-sex model’s altered terminology may also lead to the creation of potentially misleading comparisons based on the use of similar terms that have very different meanings for animal versus land plant species. Consider, for instance, the works of Till-Bottraud et al. (2005) and of Bernasconi et al. (2004) both of which compare angiosperm male gametophytes to animal sperm, which seems to assume that pollen (male gametophytic organisms) are just gametes? Or, consider the comparison of “self-fertilization” said to be done by “hermaphroditic” angiosperm sporophytes, to the self-fertilization of truly hermaphroditic animals (Jarne and Charlesworth 1993). If our goal is to make valid comparisons about aspects of sexual reproduction across taxa, the question must be asked as to whether this simple plant two-sex model is helping or hindering that effort?

In contrast, the proposed plant four-sex model would define these terms in a manner that largely follows their meanings as used for animal sexual reproduction (Table 1), perhaps clarifying matters somewhat. Notice however that in angiosperms the four-sex model would consider only the gametophytes to be male and female, and so directly comparable to male and female animals. The four-sex model would require us to regard the angiosperm sporophytic individuals as having a type of sexual reproduction not seen in animals, and so we would have to recognize the angiosperm sporophyte as having a new pair of sexual reproductive states (staminate and pistillate) (Fig. 2c). A potential benefit of adopting this new four-sex model is that it may allow us to make more valid comparisons with animals, while still recognizing how plants are indeed different from animals. Also, being rooted in the context of the alternation of generations life cycle, these three-sex and four-sex plant models may permit more valid comparisons of sexual reproductive features to be made between the various taxa of land plants by encouraging them to be done between homologous stages of the life cycle. Thus these new models challenge us to alter our thinking and that may open up new opportunities.

### Possible new perspectives from the plant four-sex model

A good model not only summarizes existing knowledge, but it also sets the framework for new questions. There have been many studies that have examined aspects of angiosperm sexual reproduction using the two-sex model. However many of these may benefit from the new perspective offered by the new four-sex model. Next just a few examples of areas of study will be presented that, if put into the framework of an angiosperm four-sex model, might open up some conceptual space which may assist our thinking.

The current two-sex model portrays the angiosperm sporophyte as the direct parent of its sporophytic offspring (the embryo in the seed), which suggests we should view the flower as involving just these two sporophytic generations. Under the four-sex model, an angiosperm flower is a larger multi-generational community, as is expected from the alternation of generations life cycle which produces distinct individuals with each generation. As Mauseth (2017) describes it, the parent angiosperm sporophyte has the male and female gametophytes as its offspring, which in turn create a primary endosperm cell as well as a zygote, which grow into endosperm and a sporophyte respectively as the gametophytes’ offspring: This new embryonic sporophyte in the seed is the grand' offspring' of the initial sporophyte which bears the flower in which these two subsequent sexual generations arise. The way in which the four-sex model permits us to view the gametophyte generation as individuals thus may alter our view of the flower into a multi-generational pageant. This illustrates, perhaps, how the reality of the alternation of generations life cycle used by angiosperm species requires us to consider all the adult organisms across its life cycle. How the original sporophyte nurtures and supports in turn both their gametophytic offspring, as well as the way the gametophytes produce the endosperm, which their parent sporophyte supplies with nutrients, so that this can be available for the gametophytes’ other progeny (the young sporophyte), which is the original sporophyte’s grand’ offspring,’ becomes a very interesting set of multi-generational interactions. Many of these interactions may be seen as altruistic and may fall under kin selection (Friedman 1995; Nowak 2006; Boomsma 2007; Bourke 2014; Dudley 2015; Bawa 2016; Placette 2020), as they occur between related individuals of different generations. This view of multi-generational cooperation in the flower would seem to be one that the four-sex model promotes, but which the current two-sex model seems to obscure as it does not fully recognize the gametophytes as players in this pageant perhaps because it does not fully accept the implications of the alternation of generations life cycle.

As another example of how the four-sex model may contribute to our thinking, consider the issue of genetic expression patterns leading to sex cell determination. Under the two-sex model in angiosperms once the type of sporogenesis is determined the “male” or “female” sex is often considered to also be established. Thus many studies of angiosperm sex determination focus on the sporophytic stage (Charlesworth 2016; Moore et al. 2016a; Sandler et al. 2018; Nelms and Walbot 2019). But under a four-sex model the issue may be expanded to consider not just what gene expressions are essential in the angiosperm sporophytic tissues to achieve proper microsporogenesis and megasporogenesis, but also what gene expressions are needed in the gametophytes to ensure formation of egg and sperm. The genetic expressions used in sporophytes may well differ from that used in gametophytes. This topic has received some attention as various studies have been done that explore gene expression and other differences between gametophytes and sporophytes, or in gametophytes across time (Bokvaj et al. 2015; Beaudry et al. 2020; Liu and Wang 2021). Some studies suggest that the development of the angiosperm gametophytes may use distinct systems of differentiation leading to gamete formation relative to the controls used for entry of some sporophytic cells into meiosis (Ranganath 2003; Zhao et al. 2017). Hoffmann and Palmgren (2013) note that there are many pollen-specific gene expressions which are repressed in the sporophytic individual. This issue has also been explored in bryophytic species (Szövényi et al. 2011; Chen et al. 2019). And various methods might be used or modified to further the exploration of this topic (Kranz and Kumlehn 1999; Brady et al. 2007; Li et al. 2019). However, how this information would fit under the current two-sex model may be problematic, as the two-sex model’s broad “male” and “female” concepts, and its tendency to obscure the gametophyte generation, may tend to influence how we view any differences found between gametophytes and sporophytes in these gene expression patterns. It might be that the new four-sex model, by accepting that both gametophytes and sporophytes are distinct sexual organisms, creates a better conceptual context for studies which hope to explore differences in gene expression patterns leading to sex cell formation in each of the four types of sexual reproductive states.

Next consider another example of how the four-sex model may aid our thinking. Pannell (2017) suggests that the control of sexual determination seems oddly to be variable across land plant groups as under the two-sex model it seems to shift from being done by the gametophytes in many homosporous plant species, to being done by sporophytes in many heterosporous plant species including the seed plants. When viewed under the three- and four-sex models this apparent shifting may well vanish: In both homosporous and heterosporous plant species the sporophytes may have sexual determination in terms of which tissues will form its sporangia and so make sexual spores, while the gametophytes may have another determination process involved in their formation of gametes. Thus it would seem more proper under the three- and four-sex models to compare homologous sexual determination processes in comparable stages of the life cycle; thus sporophytes to sporophytes, and gametophytes to gametophytes. The two-sex model, by its tendency to combine sporogenesis and gametogenesis, may obscure an examination of these sorts of issues, while the three- and four-sex models may promote such questions. There are many other issues relating to plant sexual reproduction for which the three- and four-sex models may provide new perspectives, and so may promote new lines of thinking.

## In conclusion

Here a view of aspects of sexual reproduction in the land plants from within the context of the alternation of generations life cycle has been presented in the form of two new models of plant sexual reproduction (Fig. 2a and c), which are quite different from the current plant two-sex model (Fig. 2b and d). There are several key conceptual features upon which these new models rest (Table 2). One is that the process of sporic meiosis is indeed a sexually reproductive process. This makes the plant sporophytic individuals, which carry out sporic meiosis, sexually reproductive organisms, while accepting that the plant gametophytic individuals which carry out fertilization at another point in the life cycle are also sexually reproductive. Another feature of these models is an acceptance that in the alternation of generations life cycle the sporophytes and gametophytes are indeed distinct adult multicellular individuals, making the sexual reproductive features of each plant species a collection of those that occur across all of these different types of adult organisms. Thus gametophytic and sporophytic individuals can each have their own sexual reproductive features, distinct from each other, but which then need to be summed in order to characterize the sexual reproductive features of their species as a whole. Also of importance for these new models is the limiting of the assignment of male and female sexual designations to organisms that actually make sperm and eggs, which in land plants are the gametophytes. This implies that the sporophytes are neither male nor female, but another type of sexual state entirely; one not found in animals. Another feature of these models is that mating is defined strictly as involving interactions between sperm- and egg-forming individuals, and so can be done by gametophytic individuals but not by sporophytic individuals. One implication of this is that pollination then becomes a process distinct from mating, and as something not analogous to what is seen in most animal species. Finally, the key assumption of the current two-sex model as applied to land plants is that there are only two sexual reproductive states: male and female. Here that assumption has been questioned in the new models which are presented. The proposal is made that homosporous plant species have three sexual reproductive states (Fig. 2a); male and female gametophytic states, but also a sporophytic state. And it is proposed that heterosporous plant species have four sexual reproductive states (Fig. 2c); male and female states being assigned to gametophytic individuals, and microsporangiate/antherate/staminate as well as megasporangiate/ovulate/pistillate sexual designations being assigned to sporophytic individuals. In sum, these new models are quite different from the currently accepted two-sex model, and their recognition of sporophytes and gametophytes as distinct sexually reproductive individuals may produce better clarity compared to the way the two-sex model tends to obscure one or the other generation.

**Table 2:** The meanings of concepts critical to the proposed land plant three-sex and four-sex models

Essential concept/term	Meaning under the three- and four-sex models: (meaning under the two-sex model)
Sporic meiosis	A means of sexual reproduction via the production by meiosis of sexual spores, which are distinct in ploidy from the parent sporophytic organism and capable of growing into a multicellular gametophytic organism
	(Sometimes seen as reproductive, but typically regarded as asexual in nature)
Alternation of generations	Sporophytic sexually reproductive organisms alternate with distinct gametophytic sexually reproductive organisms in a species’ life cycle
	(While sporophyte and gametophyte stages exist, typically only one is accepted as a sexually reproductive stage in a given land plant species)
Males and females	These are the gamete-forming individuals, which are the gametophytes. Sporophytes which never make gametes, are, therefore, neither male nor female
	(In homosporous land plant species the gametophytes are accepted as male and female. However, in heterosporous species these designations are often given to the sporophytic individuals, or these concepts are expanded so as to cover both sporogenesis and gametogensis across sporophytes and gametophytes)
Mating	Interactions between the sperm-forming and egg-forming individuals which precede and facilitate achievement of fertilization. Since sporophytic individuals never make eggs or sperm they do not engage in mating. Pollination is not a type of mating
	(In homosporous land plant species the gametophytes are said to have mating interactions, but in heterosporous seed plant species often pollination is viewed as being mating and is said to be done by sporophytes)
Sexual reproductive states	Homosporous land plant species have three sexual reproductive states: Sporophyte, male and female. While heterosporous land plant species have four sexual reproductive states: Microsporangiate/staminate, megasporangiate/pistillate, male and female
	(Only male and female sexual states exist)

It has long been recognized that the study of land plant sexual reproduction can be confusing, and many people have made efforts to address this by proposing revisions of the associated terminology, for instance see Jones (1918), or Cruden and Lloyd (1995). But many of these attempts base their terminology in the two-sex model, which in my view is itself the source of much of the confusion; what real difference can a new set of terminology make if plants are treated (incorrectly) as though they are just like animals? Thus, it seems that the key issue to be resolved is not the formation of new sets of terms, but rather how the alternation of generations life cycle is to be interpreted in the context of land plants. Therefore, the three- and four-sex models, based on the alternation of generations life cycle, have been presented for consideration, in the hope that they provide a more productive base on which to build than the current two-sex model. One result of adoption of these new models might be that comparisons of sexual reproductive traits seen in homosporous versus heterosporous land plant species may perhaps become clearer by promoting the comparison of homologous stages of the life cycle between each of these groups. Another may be that some of the current rather confusing redefinitions of common terms used under the two-sex model (Table 1) might be avoided. Also many of the terms used for animals species with regard to sexual reproduction will be noted to fit well in the new three- and four-sex models with regard to the plant individuals in the gametophytic generation, which is perhaps natural as both animals and plant gametophytic individuals are focused on achieving fertilization. Comparisons between gametophytic plant individuals and animals, therefore, might lead to some productive insights into the evolution of ways to achieve fertilization? It is the sporophytic plant generation that stands out as different from animals in these new models. This seems to be a natural result of the fact that plant sporophytic individuals use the process of sporic meiosis which is reproductive, while animals use gametic meiosis which is not reproductive. Which in turn is a direct consequence of the land plant life cycle being different from that of the animal life cycle. Thus, it is proposed that a close consideration of the life cycle of land plants may be key to increasing clarity in this field of study.

Whether these new models of land plant sexual reproduction account better and more clearly for the reality of the alternation of generations life cycle, and whether they provide productive new avenues for future investigations, is for the community of biologists to determine. But in doing so, we would be wise to be careful to accept plants as they are and avoid making them seem to be like animals; if our goal is to get to the truth then simplifications that obscure reality should be avoided. Adopting the three- and four-sex models of land plant sexuality may also give us a wonderful insight to a view of life in which many other eukaryotic taxa may also have more sexual states than the mere two that we poor animals typically display. If these new models are accepted, then we should share this new view of expanded sexual reproduction with our students so that when they walk under a forest canopy, and reach out to touch the trees, they can realize that they are encountering beings truly distinct from animals, not just by being different physiologically and structurally, but also by being different sexually.

In closing, a quote from Emerson (1924; pg. 182) would seem appropriate:

*“Finally, let me observe that, even though this missionary epistle to the brethren who dwell in darkness fail to convert them, it should at least afford them a somewhat unfamiliar point of attack. And, if their subsequent efforts result in my own conversion, I, at least, shall feel that I have not labored in vain.”*

### Author contribution statement

STM conceived and wrote this full article in all its parts, and is its sole author.

## Declarations

### Figure software

Figures created with Slide Write Plus for Windows, version 7.01.
